# FTO Sensitizes Oral Squamous Cell Carcinoma to Ferroptosis via Suppressing ACSL3 and GPX4

**DOI:** 10.3390/ijms242216339

**Published:** 2023-11-15

**Authors:** Ziyi Wang, Hongyu Li, Hongshi Cai, Jianfeng Liang, Yaoqi Jiang, Fan Song, Chen Hou, Jinsong Hou

**Affiliations:** 1 Department of Oral and Maxillofacial Surgery, Hospital of Stomatology, Guanghua School of Stomatology, Sun Yat-sen University, 56 Lingyuan Road West, Guangzhou 510055, China; wangzy39@mail2.sysu.edu.cn (Z.W.); lihongy5@mail2.sysu.edu.cn (H.L.); caihsh3@mail2.sysu.edu.cn (H.C.); liangjf3@mail2.sysu.edu.cn (J.L.); jiangyq8@mail2.sysu.edu.cn (Y.J.); songf6@mail2.sysu.edu.cn (F.S.); houch3@mail2.sysu.edu.cn (C.H.); 2 Guangdong Provincial Key Laboratory of Stomatology, Guanghua School of Stomatology, Sun Yat-sen University, Guangzhou 510080, China

**Keywords:** N6-methyladenosine, FTO, ferroptosis, oral squamous cell carcinoma

## Abstract

Ferroptosis is a newly established form of regulated cell death characterized by intracellular lipid peroxidation and iron accumulation that may be a promising cancer treatment strategy. However, the function and therapeutic value of ferroptosis in oral squamous cell carcinoma (OSCC) remain inadequately understood. In the present study, we investigated the biological role of the fat mass and obesity-associated gene (FTO) in ferroptosis in the context of OSCC. We found that OSCC had greater potential for ferroptosis, and FTO is associated with ferroptosis. Furthermore, higher FTO expression sensitized OSCC cells to ferroptosis in vitro and in vivo. Mechanistically, FTO suppressed the expression of anti-ferroptotic factors, acyl-CoA synthetase long-chain family member 3 (ACSL3) and glutathione peroxidase 4 (GPX4), by demethylating the m^6^A modification on the mRNA of ACSL3 and GPX4 and decreasing their stability. Taken together, our findings revealed that FTO promotes ferroptosis through ACSL3 and GPX4 regulation. Thus, ferroptosis activation in OSCC with high FTO levels may serve as a potential therapeutic target.

## 1. Introduction

Cancer remains the foremost health concern globally, with oral cancer constituting 2% of all malignancies. Over 370,000 new cases and more than 170,000 deaths were reported in 2020 alone according to the International Agency for Research on Cancer 2020 report [1]. Oral squamous cell carcinoma (OSCC) is the most prevalent form of oral cancer, and it is largely attributed to tobacco and/or alcohol consumption and areca chewing [2,3,4,5]. Despite advances in diagnostic techniques and therapies, patients often present at an advanced stage [2]. The high recurrence rate and frequent lymphatic metastasis contribute to the relatively low five-year survival rate of OSCC [6]. Currently, surgery is the primary treatment for OSCC; it is often supplemented by chemotherapy and radiotherapy, which occasionally yield side effects [7]. Immune checkpoint inhibitors (ICIs) have recently emerged as a promising strategy for cancer treatment, including for OSCC [8,9,10]. However, the outcomes have been inconsistent and not all patients benefited from ICIs [8,11]. Therefore, a thorough understanding of the molecular mechanisms underlying OSCC is imperative for developing new and effective treatments, given the limitations of the current treatments and the intense aggressiveness and frequent metastasis of this disease.

N6-methyladenosine (m^6^A) is the most common internal modification of RNA in eukaryotic cells, and it plays a pivotal role in regulating mRNA stability, splicing, translation, and localization [12,13]. The m^6^A modification process, regulated by enzymes such as m^6^A methyltransferases (writers), demethylases (erasers), and binding proteins (readers), is reversible. These enzymes catalyze the generation and elimination of m^6^A modification and recognize specific m^6^A sites. The dysregulation of m^6^A modification can lead to tumorigenesis, invasion, and metastasis in various cancers [14,15]. The fat mass and obesity-associated gene (FTO), the first demethylase of m^6^A modification identified, has been found to regulate tumor phenotypes such as oncogenesis, proliferation, and invasions in several cancers [16,17]. FTO has been found to regulate the immune response of arecoline-exposed oral cancer by targeting the m^6^A modification of PD-L1 [18]. Our previous studies revealed that FTO regulates autophagy and malignant progression in OSCC by mediating eukaryotic translation initiation factor gamma 1 (eIF4G1) mRNA m^6^A modification, establishing FTO as a potential therapeutic target for OSCC [19].

Ferroptosis is a unique, iron-dependent form of regulated cell death that was first identified by Dr. Brent R. Stockwell in 2012 [20]. It is characterized by lipid peroxidation, an accumulation of reactive oxygen species (ROS), and the dysregulation of the cellular antioxidant system, via several metabolic pathways [21,22]. Ferroptosis has been implicated in various pathophysiological processes, including acute kidney injury [23], heart disease [24], neurodegenerative disease [25], and cancer [22,26]. Given the reliance of cancer cells on iron and the hallmark of apoptosis resistance, ferroptosis has emerged as a promising strategy in cancer treatment [21,27,28,29,30,31,32]. The relationship between ferroptosis and m^6^A modification has garnered significant attention in cancer research [33,34]. Recent studies have implied that FTO might play a role in ferroptotic cell death [35,36,37]. However, the intrinsic mechanism of ferroptosis and FTO in OSCC remains inadequately understood. In the present study, FTO is associated with the sensitivity of ferroptosis in OSCC. The mechanism underlying FTO’s promotion of ferroptosis and the potential mRNA targets of FTO were subsequently investigated both in vivo and in vitro by establishing an FTO-overexpression model and a nude mouse xenograft model. Given FTO’s tumorigenic effect and the emerging role of ferroptosis treatment, the FTO–m^6^A axis may serve as a therapeutic target for oral squamous cell carcinoma.

## 2. Results

### 2.1. Ferroptosis in OSCC Is Correlated with the m^6^A Modification

**Figure 1:**
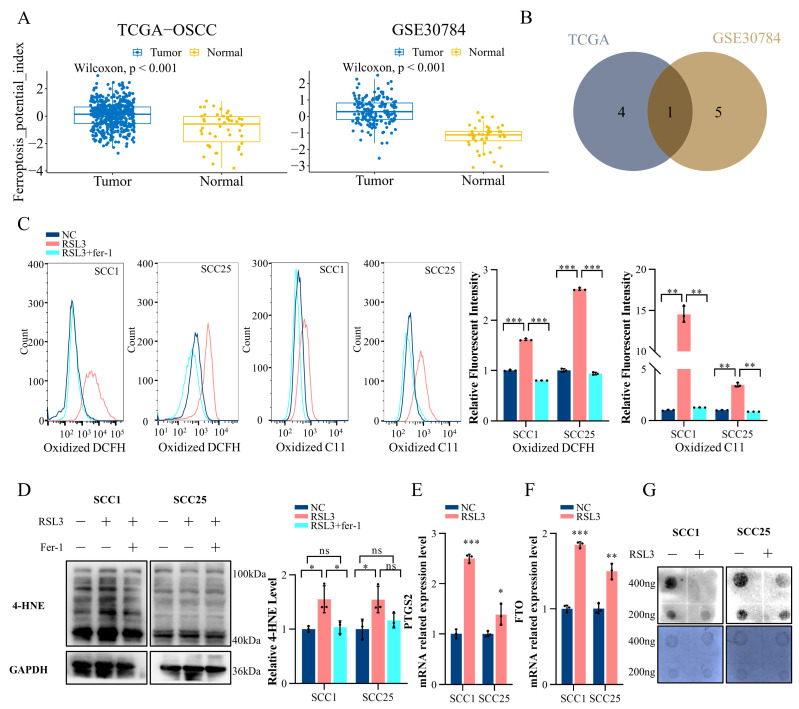
Ferroptosis in OSCC is correlated with the m^6^A modification. (**A**) The ferroptosis potential index (FPI) was evaluated in a OSCC sample in The Cancer Genome Atlas (TCGA) database and GSE30784 data set. (**B**) Venn diagram showing the common m^6^A-related protein correlated with the FPI in TCGA-OSCC and GSE30784. (**C**) Reactive oxygen species and lipoperoxidation level of RSL3/RSL3 + fer-1-treated OSCC cells measured by flow cytometry. ** *p* < 0.01, *** *p* < 0.001. (**D**) Western blot detecting the 4-HNE level of RSL3/RSL3 + fer-1 treated-OSCC cells. GAPDH serving as the control. * *p* < 0.05. (**E**,**F**) RT-qPCR detecting the change in PTGS2 (**E**) and FTO (**F**) mRNA levels after RSL3 application to OSCC cells. * *p* < 0.05, ** *p* < 0.01, *** *p* < 0.001. (**G**) Dot blot assay revealing the changes in the m^6^A level in RSL3-treated OSCC cells.

Our prior research has established a profound link between m^6^A modification and the progression of oral squamous cell carcinoma (OSCC). The ferroptosis potential index (FPI) is a calculated metric that uses RNA-seq data to evaluate the potential level of ferroptosis. We examined 354 OSCC samples extracted from 546 samples of head and neck squamous cell carcinoma patients from The Cancer Genome Atlas (TCGA) and 212 samples collected from GSE30784. Our findings indicate that the FPI in OSCC patients is significantly elevated compared to that in normal tissue (Figure 1A). Upon conducting a correlation analysis, we discovered that the m^6^A demethylase FTO exhibits the most substantial correlation in the TCGA-OSCC cohort and GSE30784 data set when compared with other major m^6^A modification writers, erasers, and readers (Figure 1B).

To further substantiate the relationship between m^6^A modification and ferroptosis, we established a ferroptosis cell model using RSL3, a ferroptosis inducer. As the rise of reactive oxygen species (ROS) and lipid peroxidation are considered significant in ferroptosis onset [38,39], we measured the levels of ROS and lipid peroxidation using DCFH-DA and BIODIPY-C11 probes, respectively, after treating SCC1 and SCC25 cells with RSL3, with or without ferroptosis inhibitor fer-1, for 24 h. After treatment and staining with the probes, the cells were collected. Compared with non-RSL3-treated cells, a significant increase in ROS and lipid peroxidation levels was observed in RSL3-treated cells. Meanwhile, the ferroptosis inhibitor fer-1 restored the changes, showing a much lower ROS and lipid peroxidation level in the RSL3 + fer-1-treated group (Figure 1C). As one of the final by-products of lipid oxidation, 4-hydroxynonenal (4-HNE) is considered a marker of ferroptotic lipid peroxidation. Similarly, we observed a higher 4-HNE level in RSL3-treated cells (Figure 1D), and 4-HNE levels in the RSL3 + fer-1 group decreased compared with RSL3-treated cells. As part of genetic changes of ferroptosis, the prostaglandin endoperoxide synthase2 (PTGS2) mRNA level was elevated in the RSL3 group (Figure 1E). Subsequently, changes in FTO expression levels were detected in two different OSCC cell lines under ferroptosis conditions (Figure 1F). In the ferroptosis state, m^6^A levels decreased In accordance with the FTO change (Figure 1G). Collectively, these findings suggest that a decrease in m^6^A levels is associated with ferroptosis, and FTO plays a pivotal role in the occurrence of ferroptosis in OSCC.

### 2.2. Upregulation of FTO Expression Enhances Ferroptosis in OSCC Cells

**Figure 2:**
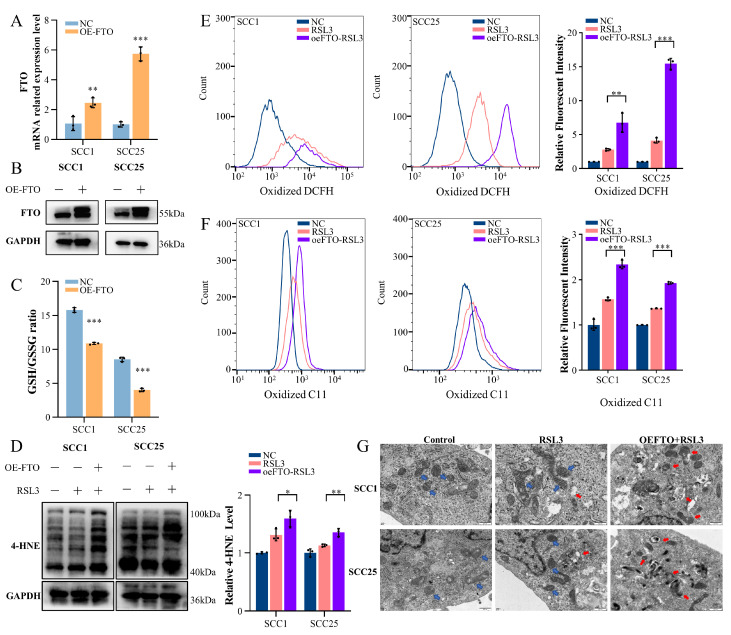
Upregulation of FTO expression enhances ferroptosis in OSCC cells. (**A**,**B**) The efficiency of FTO overexpression was demonstrated by RT-qPCR (**A**) and Western blot (**B**) analyses of the SCC1 and SCC25 cell lines. ** *p* < 0.01, *** *p* < 0.001. (**C**) GSH/GSSG ratio of SCC1 and SCC25 cells with or without FTO overexpression. *** *p* < 0.001. (**D**) Western blot 4-HNE assay after ferroptosis induction, with more 4-HNE produced in FTO-overexpressing cells. * *p* <0.05, ** *p* < 0.01. (**E**,**F**) Reactive oxygen species (**E**) and lipoperoxidation (**F**) levels of control, RSL3, and FTO overexpression + RSL3 groups in UM-SCC1 and SCC25 cells measured via flow cytometry. ** *p* < 0.01, *** *p* < 0.001. (**G**) The morphological changes of mitochondria in the control, RSL3, and FTO overexpression + RSL3 groups were demonstrated by a transmission electron microscope in SCC1 and SCC25 cell lines. Blue arrows point to the normal mitochondria and red arrows point to the shrunken mitochondria.

To verify that the induced ferroptosis in RSL3-treated cells is associated with an increased FTO level, we generated a lentiviral overexpression plasmid for FTO to enhance the endogenous FTO expression in SCC1 and SCC25 cells, and real-time quantity-PCR (RT-qPCR) and Western blotting were conducted to evaluate the effectiveness (Figure 2A,B). However, whether stable FTO-overexpressing cells are more susceptible to ferroptosis needs to be further explored. Glutathione (GSH) is the most important intracellular antioxidant, protecting cells from oxidative damage. The GSH/GSSG ratio is an important marker of the intracellular oxidation stage, which could reflect sensitivity to ferroptosis [40]. A lower GSH/GSSG ratio was observed in stable FTO-overexpressing cells (Figure 2C), which may indicate a stronger sensitivity. On the other hand, a 4-HNE assay showed that when treated with the same RSL3 concentration for 24 h, stable FTO-overexpressing cells displayed a higher 4-HNE level than the control cells (Figure 2D). In agreement with the 4-HNE change, similar changes were observed in ROS and lipid peroxidation detection. Stable FTO-overexpressing cells demonstrated a substantial rise in both ROS and lipid peroxidation compared with the control group after ferroptosis induction (Figure 2E,F). Mitochondrial changes are a key morphological feature of ferroptosis. Utilizing transmission electron microscopy, we observed that RSL3-treated FTO-overexpressing cells exhibited more shrunken mitochondria than RSL3-treated control cells (Figure 2G). These results suggest that enhancing FTO expression promotes ferroptosis in OSCC cells.

### 2.3. FTO Enhances OSCC Cell Ferroptosis In Vivo

**Figure 3:**
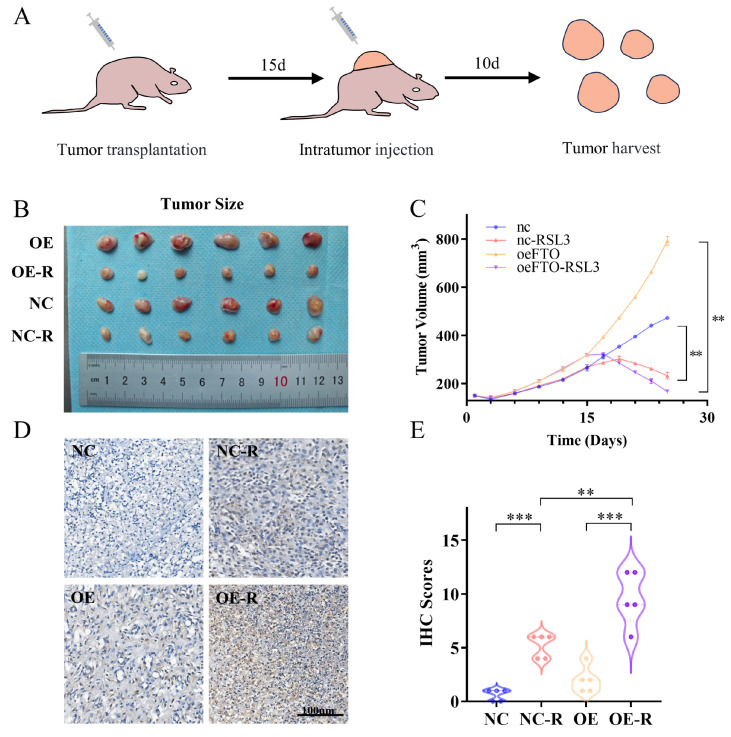
FTO enhances OSCC cell ferroptosis in vivo. (**A**) Specific strategy and timeline of animal experiments. (**B**,**C**) The tumor size was recorded every three days before the intratumoral RSL3 injection and every two days after the RSL3 injection. ** *p* < 0.01. (**D**,**E**) The immunohistochemistry of the tumor was performed to measure the 4-HNE level of each group. ** *p* < 0.01, *** *p* < 0.001. IHC, immunohistochemistry.

Having demonstrated the ferroptosis-promoting role of FTO in vitro, we sought to further validate its effect using an in vivo model. We inoculated both control and stable FTO-overexpressing SCC25 cells subcutaneously into immune-deficient athymic nude mice (Figure 3A). We selected a dose of 1.2 × 10^7^ cells for our experiments. At a dose of 10 mg/kg body weight, RSL3 was injected intratumorally every other day. After the RSL3 injection, we observed a significant suppressive effect of RSL3 on tumor growth in the oeFTO–RSL3 group compared with the oeFTO group. A similar suppressive effect was recorded between the control group and the control–RSL3 group. Alongside that, the suppressive effect was more frequently observed in the oeFTO–RSL3 group than in the control–RSL3 group (Figure 3B,C). The 4-HNE immunohistochemistry staining was conducted to evaluate the level of lipid peroxidation. These results show that the oeFTO group presented moderate 4-HNE staining while the control group did not. Moreover, when treated with RSL3, lipid peroxidation was more pronounced in stable FTO-overexpressing cells compared with control cells (Figure 3D,E). These results indicate that increased FTO expression could promote ferroptosis in vivo and that stable FTO-overexpressing cells were more sensitive to ferroptosis.

### 2.4. FTO Promotes Ferroptosis in OSCC Cells by Downgrading ACSL3 and GPX4 Expression

**Figure 4:**
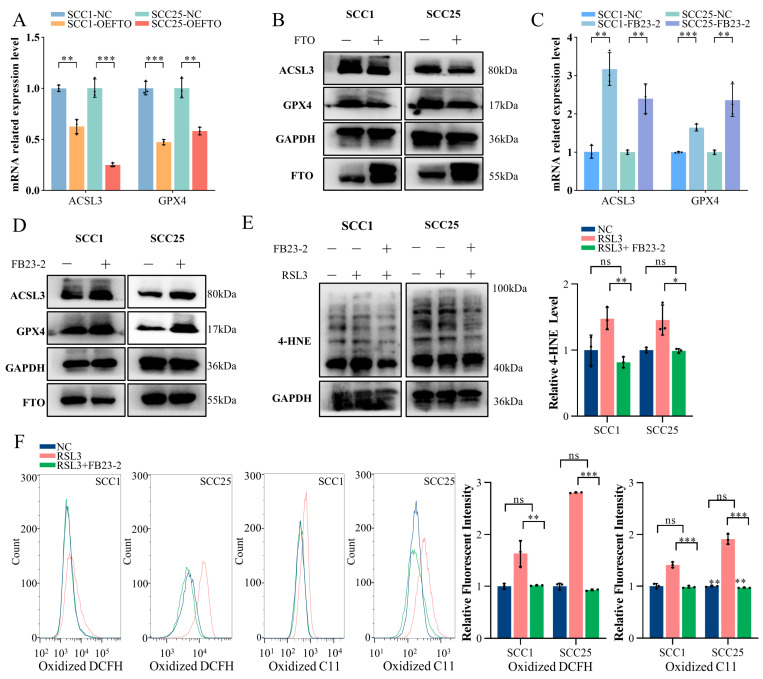
FTO promotes ferroptosis in OSCC cells by downgrading the expression of ACSL3 and GPX4. (**A**,**B**) The expression level of ACSL3 and GPX4 in the control and stable FTO-overexpressing groups was demonstrated using RT-qPCR (**A**) and Western blot (**B**) analyses for the OSCC cell lines. ** *p* < 0.01, *** *p* < 0.001. (**C**) The mRNA level of ACSL3 and GPX4 in the control and FB23-2-treated groups was demonstrated using RT-qPCR. ** *p* < 0.01, *** *p* < 0.001. (**D**) Western blot was performed to detect the expression change of ACSL3 and GPX4 in FB23-2-treated SCC1 and SCC25 cells. (**E**) A Western blot 4-HNE assay displayed after ferroptosis induction, with less 4-HNE produced in FB23-2-treated cells than the control. * *p* < 0.05, ** *p* < 0.01. ns means not significant. (**F**) Reactive oxygen species and lipoperoxidation levels of the control, RSL3, and FB23-2 + RSL3 groups in SCC1 and SCC25 cells measured via flow cytometry. ** *p* < 0.01, *** *p* < 0.001. ns means not significant.

To elucidate the precise mechanism by which FTO promotes ferroptosis, we conducted an RT-qPCR to examine changes in the major drivers and suppressors of ferroptosis. The results indicate that the levels of acyl-CoA synthetase long-chain family member 3 (ACSL3) and glutathione peroxidase 4 (GPX4) were significantly reduced in FTO-overexpressing cells (Figure 4A). In line with mRNA findings, these proteins also appeared to be negatively regulated by FTO (Figure 4B, Supplementary Figure S1A).

To further investigate the role of FTO’s m^6^A demethylase function, we applied FTO inhibitor FB23-2, which is a small molecular compound that selectively inhibits the m^6^A demethylase activity of FTO. In vitro, the mRNA and protein expression of ACSL3 and GPX4 were elevated compared to the control group in SCC1 and SCC25 cells after incubating with 2 µM FB23-2 for 48 h (Figure 4C,D, Supplementary Figure S1B). Additionally, after silencing FTO, the expression of ACSL3 and GPX4 in SCC1 and SCC25 cells showed similar promotion (Supplementary Figure S1C). These results demonstrate that the FTO downregulation of ACSL3 and GPX4 expression may occur through its m^6^A demethylase activity. Moreover, an increased GSH/GSSG ratio was discovered after applying FB23-2 to SCC1 and SCC25 cells (Supplementary Figure S1D). To further confirm whether the FTO inhibitor FB23-3 could revise the FTO function on ferroptosis, RSL3 was used after FB23-2 treatment. As expected, OSCC cells became less responsive to ferroptosis after treatment with FB23-2. The 4-HNE Western blot demonstrated a lower 4-HNE level in FB23-2-treated cells after the application of RSL3 (Figure 4E). As observed for cell death, intracellular ROS and lipid peroxidation levels were reduced by FB23-2 (Figure 4F). Taken together, these findings suggest that FTO sensitizes cells to ferroptosis by reducing ACSL3 and GPX4 expression.

### 2.5. FTO Demethylates the m^6^A Modification on ACSL3 and GPX4 mRNA

**Figure 5:**
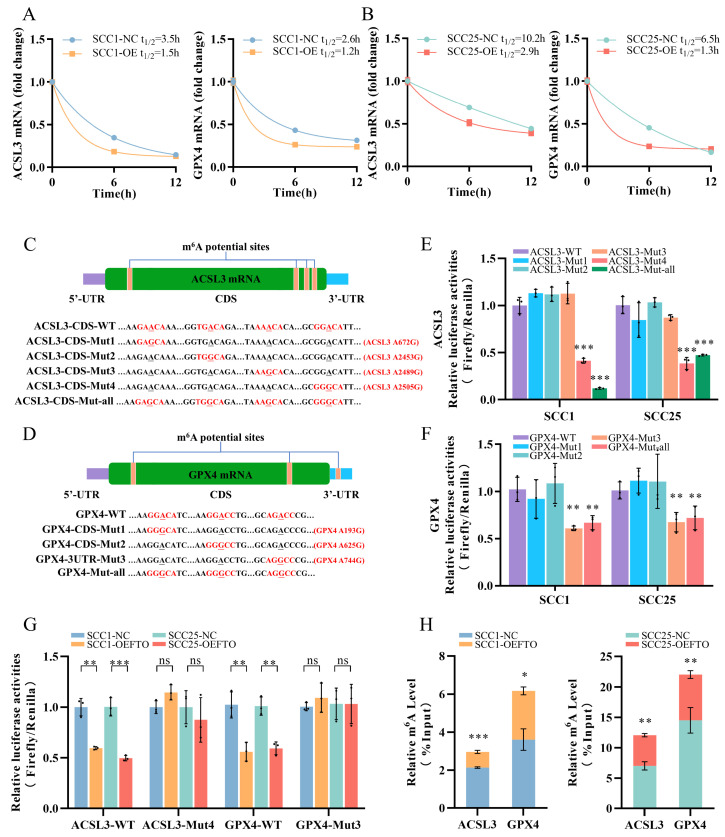
FTO demethylates the m^6^A modification on the mRNA of ACSL3 and GPX4. (**A**,**B**) RNA stability assay showing the mRNA half-life (t1/2) in SCC1 (**A**) and SCC25 (**B**) cells with or without FTO overexpression. (**C**,**D**) Schematic representation of the positions of m^6^A potential sites with ‘very high confidence’, and mutations in ACSL3 (**C**) and GPX4 (**D**) mRNA are shown. The predicted m^6^A motifs and their mutations were highlighted in red, respectively. The underlined characters represented the potential m^6^A modifications and their mutations. “RRAC, R = G/A” is the shared m^6^A potential motif of ACSL3 and GPX4 mRNAs. CDS, coding sequences. UTR, untranslated regions. (**E**,**F**) Dual-luciferase reporter plasmas were generated to confirm the m^6^A potential sites. Each of the ‘very high confidence’ sites of ACSL3 (**E**) and GPX4 (**F**) mRNA were mutated and transferred to the reporter plasmas. Wild-type (WT) and mutated reporter plasmas were transfected into SCC1 and SCC25 cells. The translation efficiency of ACSL3/GPX4 was defined as the quotient of the reporter protein production (firefly/renilla). ** *p* < 0.01, *** *p* < 0.001. (**G**) A dual-luciferase reporter experiment was conducted to confirm the m^6^A sites recognized by FTO in the control and stable FTO-overexpressing cells. ** *p* < 0.01, *** *p* < 0.001. ns means not significant. (**H**) Me-RIP qPCR demonstrated that the m^6^A modification level of the confirmed site was decreased in SCC1 and SCC25 cells with stable FTO-overexpressing. * *p* < 0.05, ** *p* < 0.01, *** *p* < 0.001.

The primary function of FTO is to demethylate m^6^A modifications on mRNA. We hypothesized that the absence of m^6^A modification triggers the degradation of ACSL3 and GPX4 mRNA. We then used actinomycin D to inhibit RNA synthesis to determine the decay rate of ACSL3 and GPX4 mRNA. The half-life of ACSL3 and GPX4 mRNA was significantly shorter in FTO-overexpressing cells, indicating a decrease in their mRNA stability (Figure 5A,B). However, it remained uncertain whether FTO recognizes and removes the N6-methyladenosine modification on the mRNA of ACSL3 and GPX4. To address this, the online database SRAMP was explored to identify potential m^6^A sites within ACSL3 and GPX4 mRNA. For further validation, we selected potential m^6^A sites with ‘very high confidence’ scores to investigate the interaction between FTO and these anti-ferroptotic mRNA (Supplementary Figure S2A,B). These m^6^A sites were located near or within the mRNA’s regulatory region. Additionally, the shared m^6^A motif of ACSL3 and GPX4 mRNAs, “RRAC, R = G/A”, is considered a conserved m^6^A motif across species, lending credibility to the SRAMP results (Figure 5C,D).

Subsequently, we performed dual-luciferase reporter and mutagenesis assays in SCC1 and SCC25 cells. We created dual-luciferase reporter plasmas containing firefly luciferase, followed by wild-type and mutated sequences shown in the figures (Figure 5C,D). The Mut4 sequence of ACSL3 and the Mut3 sequence of GPX4 exhibited a significant decrease in the firefly/renilla ratio, confirming that the primary m^6^A site of ACSL3 mRNA is located in its Mut4 sequence, and the primary m^6^A site of GPX4 mRNA is located in its Mut3 sequence (Figure 5E,F). To further verify whether FTO demethylates the m^6^A modification at these two specific sites, reporter plasmas containing wild-type and mutated sequences (ACSL3 WT/Mut4 and GPX4 WT/Mut3) were transfected into OSCC cells with or without FTO overexpression, respectively. The results demonstrated that, in FTO-overexpressing cells, the firefly/renilla ratio dropped in both wild-type groups, whereas in the mutated group, there was no significant difference (Figure 5G). For further confirmation, n6-methylated RNA immunoprecipitation (MeRIP) was performed by using m^6^A antibodies to precipitate the fragmented RNA from SCC1 and SCC25 cells. MeRIP-qPCR revealed a decrease in m^6^A methylation levels in ACSL3 and GPX4 mRNA in the FTO-overexpressing cells (Figure 5H). These findings suggest that FTO recognized the m^6^A sites and demethylated m^6^A modifications on ACSL3 and GPX4 mRNA, leading to a faster decay rate and shorter half-life.

## 3. Discussion

As of now, N6-methyladenosine (m^6^A) modification is the most prevalent internal RNA post-transcriptional modification among over 100 known chemical modifications [41,42]. Generally, m^6^A modification has an anticancer or protumoral role in the genesis and malignancy of various tumors [13,14,15], including bladder cancer [43], ovarian cancer [44], hepatocellular carcinoma [45], and oral squamous cell carcinoma [19]. Given the significant role of m^6^A modification in tumor progression, there has been an increased focus on targeting m^6^A methylation and its associated proteins, such as FB23-2, a small molecule-targeting FTO in acute myeloid leukemia [46]. Numerous studies have underscored the crucial roles m^6^A methylation plays in drug resistance [47,48], radiotherapy tolerance [49,50], and programmed cell death [33,51]. 

Ferroptosis, a novel form of programmed cell death distinct from apoptosis, necrosis, or autophagy, is an antitumor mechanism that plays a crucial role in eliminating malignant cells [21]. The primary characteristics of ferroptosis include lipid peroxidation and iron ion accumulation, accompanied by mitochondrial membrane density compression, reduction or disappearance of mitochondrial cristae, and rupture of the outer mitochondrial membrane [39]. These changes are closely associated with the dysfunction of GPX4. This intricate process involves a series of signals from various pathways, enabling the regulation at different stages, particularly in the realm of epigenetics.

Recent studies have begun to unravel the complex relationship between various m^6^A-related proteins and ferroptosis. Solute carrier family 7 member 11 (SLC7A11), a subunit of the system Xc-, can be mediated by methyltransferase 3 (METTL3), enhancing ferroptotic resistance and promoting apoptosis and proliferation in lung adenocarcinoma [52]. Similarly, m^6^A-mediated system Xc- involvement in ferroptosis has been observed in glioblastoma [53], thyroid cancer [54], and hepatoblastoma [34]. Additionally, m^6^A reader proteins have also been implicated in regulating ferroptosis. In lung adenocarcinoma (LUAD), both SLC3A2 and SLC7A11 were identified as the targets of YTH n6-methyladenosine RNA binding protein C2 (YTHDC2) [55,56]. An increase in YTHDC2 could induce ferroptosis in LUAD by suppressing system Xc^−^. Insulin-like growth factor 2 mRNA binding protein 3 (IGF2BP3) was found to desensitize cells to ferroptosis by down-regulating several key anti-ferroptotic factors, including GPX4, ACSL3, and FTH1 in lung adenocarcinoma [57]. Current research on m^6^A-regulated ferroptosis primarily focuses on the effects of m^6^A writers and readers in disrupting lipid peroxidation and antioxidative processes. However, there is a scarcity of studies exploring the role of m^6^A erasers and their fundamental correlation with ferroptosis in cancer cells.

Our previous study has identified a close relationship between m^6^A methylation and oral squamous cell carcinoma malignancy [19]. The fat mass and obesity-related gene, FTO, is a member of the ALKB family of nucleic acid demethylases, preferentially catalyzing m^6^A sites in RNA [58]. FTO has been shown to be upregulated in various malignant neoplasms, playing a role in promoting carcinogenesis [59]. FTO is significantly overexpressed in acute myeloid leukemia and encourages malignancy progression by reducing m^6^A modification levels and regulating the expression of ankyrin repeat and socs box containing 2 (ASB2) and retinoic acid receptor alpha (RARA) [60]. Another study found that FTO expression was significantly increased in human gastric cancer, correlating closely with clinical prognosis and patient survival rates. Furthermore, silencing FTO decreased the proliferation, migration, and invasion of gastric cancer cells [61]. These findings suggest that FTO upregulation is associated with poor prognosis in cancer patients.

In the present study, we examined the relationship between the ferroptosis potential index and m^6^A-related proteins, concluding that FTO has a strong correlation with ferroptosis. Given that RSL3 has been proven to induce ferroptosis, we established an RSL3-induced ferroptosis model in OSCC cells. Previous research observed higher proliferation and metastasis rates after FTO overexpression in OSCC cells. Some studies have suggested that cancer cells resistant to conventional therapies or with a high propensity to metastasize may be particularly susceptible to ferroptosis [62,63]. As FTO expression increased, ACSL3 and GPX4 were consequently downregulated.

Polyunsaturated fatty acids (PUFAs) play a critical role in inducing ferroptosis since the multi-unsaturated chemical structure increases the possibility of lipid ROS affection. Contrastingly, monounsaturated fatty acids (MUFAs) have the opposite effect [22,26]. ACSL3 is a key factor in assembling membrane phospholipids with MUFA, an important ferroptosis suppressor [64]. Researchers have reported that ACSL3 functions as an anti-ferroptotic factor. In our study, OSCC cells were more sensitive to ferroptosis with the downregulation of ACSL3. 

Glutathione peroxidase 4, also known as GPX4, is the fourth member of the glutathione peroxidase family. Compared to other family members, GPX4 has a strong preference for lipid hydroperoxides and directly protects cells against membrane lipid peroxidation in concert with GSH [65,66]. Therefore, GPX4 is central to ferroptosis inhibition and has been a major focus with regard to inducing ferroptosis to combat cancer. Lower GPX4 expression, along with a lower GSH/GSSG ratio, indicates that cells might encounter oxidization stress, contributing to the ferroptosis potential. 

In summary, the present results demonstrated that FTO expression functions as a ferroptosis promoter by downregulating the anti-ferroptotic factors ACSL3 and GPX4. Furthermore, these results suggest that ferroptosis may offer a potential therapeutic approach for OSCC with higher FTO levels.

## 4. Materials and Methods

### 4.1. Data Collection and Analysis, and Ferroptosis Potential Index Calculation

RNA-seq data, clinical data, and follow-up data of HNSCC patients in TCGA were downloaded from the GDC website. A total of 354 samples were identified as OSCC and their normal samples. RNA-seq count data were converted to log2 (TPM + 1) values for further analysis. The ferroptosis potential index (FPI) was calculated to indicate the ferroptosis level among the patients in the TCGA-OSCC cohort and GSE30784 data set. The FPI was the normalized difference between the enrichment score of the ferroptosis positive gene set (LPCAT3, ACSL4, NCOA4, ALOX15, GPX4, SLC3A2, SLC7A11, NFE2L2, NOX1, NOX3, NOX4, NOX5) and the negative gene set (FDFT1, HMGCR, COQ10A, COQ10B). The enrichment score of both gene sets was calculated using single-sample gene set enrichment analysis (ssGSEA) with R package ‘GSVA’ [67]. Correlation analysis and visualization were performed employing the R package ‘ggstatplots’. The Venn diagram was constructed using jvenn (https://jvenn.toulouse.inrae.fr/app/index.html, accessed on 14 September 2023) [68].

### 4.2. Cell Line Culture

The human OSCC cell line SCC25 was purchased from the American Type Culture Collection (ATCC). UM-SCC1 cells were obtained from the University of Michigan. SCC25 cells were cultured with a DMEM/F12 (Gibco, New York, NY, USA) medium containing 10% fetal bovine serum (FBS, WISENT, Montreal, QC, Canada) and 400 ng/mL hydrocortisone (MACKLIN, Shanghai, China). SCC1 cells were grown in the DMEM (Gibco, New York, NY, USA) medium containing 10% FBS. The cells were cultured at 37 °C in a humidified 5% CO_2_ incubator.

### 4.3. Construction of Stable Gene Expression Cell Lines

For lentivirus packaging, 293T cells were used. The overexpression FTO plasmid or negative control plasmid and lentiviral packaging plasmids psPAX2 and pMD2.G were co-transfected into 293T cells with lipofectamine 3000 transfection reagent (Invitrogen, Carlsbad, CA, USA) for 12 h. The lentivirus supernatant was collected after transfection for 36 h. The UM-SCC1 and SCC25 cells were seeded in a 6-well plate and cultured with lentivirus particles and 10 μg/mL polybrene (H8761, Solarbio, Beijing, China). Cell selection was performed by G418 for 14 days after lentivirus infection. Quantitative real-time PCR reaction and Western blot were used to detect the expression efficiency of FTO.

### 4.4. Real-Time Quantity-PCR

RNAzol (MRC, Cincinnati, OH, USA) was used to extract total RNA from OSCC cells. Complementary DNA strands were synthesized using the Hifair III 1st Strand cDNA Synthesis kit (Yeasen, Shanghai, China), according to the manufacturer’s instructions. The reverse transcription reactions were run at 42 °C for 2 min to remove genome DNA, followed by 25 °C for 5 min, 55 °C for 15 min, and 85 °C for 5 min for reverse transcription. Quantitative real-time PCR reactions were performed with LightCycler 480 II (Roche, Basel, Switzerland) using the SYBR Green Master Mix (Yeasen, Shanghai, China), following the manufacturer’s instructions. The PCR reactions were run at 95 °C for 5 min, followed by 40 cycles of 95 °C for 10 s, 60 °C for 20 s, and 72 °C for 20 s, with a final cycle of 95 °C for 15 s, 60 °C for 60 s, and 95 °C for 15 s as the melting curve. Gene expression was calculated via 2^−ΔΔCt^ normalized to β-ACTIN. The primer sequences that were used are listed in Table 1.

### 4.5. Western Blot

The cells were lysed with a mix of RIPA buffer (CWbio, Taizhou, China) and a protease inhibitor cocktail for 15 min on ice, and centrifuged at 13,000× *g* for 20 min at 4 °C. Then, the total protein was collected, and the concentration was measured using a BCA protein assay kit. After denaturation, approximately 20 µg of the total protein was subjected to 10% SDS-PAGE and transferred to PDVF membranes (ISEQ00010, Millipore, Billerica, MA, USA). Primary antibodies were incubated for 14 h after the blocking of nonspecific binding of the membranes by 5% bovine serum albumin. Primary antibodies included anti-ACSL3 (Abcam, Cambridge, UK, 1:2500), anti-FTO (Abcam, Cambridge, UK, 1:5000), anti-GPX4 (Abcam, Cambridge, UK, 1:3000), anti-4-HNE (Abcam, Cambridge, UK, 1:2500), and anti-GAPDH (Servicebio, Wuhan, China, 1:1000) (Table 2). The membranes were conjugated to a horseradish peroxidase (HRP) secondary antibody at room temperature for 1 h, and the bands were exposed to a chemiluminescent HRP substrate (WBKLS0500, Millipore, Billerica, MA, USA). The chemiluminescence images were recorded with the ChemiDoc Imaging System (BIO-RAD, Hercules, CA, USA). A quantitative analysis of the results was conducted using Image Lab afterward.

### 4.6. m^6^A Dot Blot Assay

To validate the changes in m^6^A levels in RSL3-treated OSCC cells before and after, an m^6^A dot blot was performed. The total RNA was denatured by heating at 90 °C for 3 min and spotted onto a nylon transfer membrane (Solarbio, Beijing, China). The membrane was crosslinked using ultraviolet (125 mJ/cm^2^). After blocking and incubation with the m^6^A primary antibody (Abcam, Cambridge, UK, 1:5000) overnight at 4 °C, HRP-conjugated secondary antibody incubation was conducted. Then, the membrane was visualized via the chemiluminescence system (Bio-Rad, Hercules, CA, USA). After visualization, 0.02% methylene blue (MB) in 0.3 M sodium acetate buffer (pH 5.2) was used to stain the nylon membrane to ensure consistency in the RNA amount on the membrane of the samples.

### 4.7. Transmission Electron Microscopy (TEM)

According to a previous study, the morphological changes of OSCC cell lines ferroptosis were conducted via transmission electron microscopy [22,26]. In brief, SCC1 and SCC25 cells (120,000 cells/well) were inoculated into 6-well plates and treated with RSL3. Then, the cells were collected and pre-fixed by 2% glutaraldehyde. TEM images were captured with a JEM-1400Flash transmission electron microscopy (JEOL, Tokyo, Japan).

### 4.8. ROS and Lipid Peroxidation Detection

OSCC cells were seeded into 6-well plates and exposed to RSL3 for 24 h. DCFH-DA (APPLYGEN, Beijing, China) was used for ROS detection, and a BODIPY-C11 (Invitrogen, Carlsbad, CA, USA) probe was applied to measure the level of lipid ROS. After staining with DCFH-DA or BODIPY-C11 probes for 30 min, the cells were washed, harvested, and then measured via BD LSRFortessa flow cytometer (BD Biosciences, San Jose, CA, USA). The mean fluorescent intensity (MFI) was used for further analysis.

### 4.9. Tumor Xenograft and Immunohistochemistry

Female BALB/C nude mice (age, 4 weeks) were randomly distributed into four groups, the NC group, the NC + RSL3 group, the oeFTO group, and the oeFTO + RSL3 group (*n* = 6 per group). To establish the cell line-derived subcutaneous transplanted model, approximately 1.2 × 10^7^ SCC25 cells per mouse, diluted in 150 μL of phosphate-buffered saline, were injected subcutaneously into the right posterior flanks. Two weeks after tumor transplantation, RSL3 was injected in a manner of 10 mg/kg intratumorally every other day. The transplanted tumors were measured every 3 days to observe tumor growth before the intratumoral RSL3 injection and every 2 days after the RSL3 injection. The tumor volume (V) was calculated using the formula V = 0.5 × (length × width^2^). After treatment for 10 days, the tumor xenografts were harvested and fixed for immunohistochemistry. This animal experiment was approved by the Sun Yat-Sen University’s Animal Experiment Ethics Committee. Immunohistochemistry on the tumor xenograft tissue was performed according to our previously described protocol [69]. The 4-HNE immunohistochemistry staining scores were calculated according to the stain intensity and staining sections. The staining intensity was scored as 0 (negative staining), 1 (weak staining), 2 (moderate staining), and 3 (strong staining). The staining sections were scored as 1 (cells with <10% staining), 2 (cells with 10–49% staining), 3 (cells with 50–74% staining), and 4 (cells with 75–100% staining). The final score was defined as the staining intensity score multiplied by the staining section score.

### 4.10. GSH/GSSG Assay

To measure the GSH/GSSG ratio after enhancing and suppressing FTO expression, a GSH/GSSG assay was performed using a GSH and GSSG Assay Kit (Beyotime, Shanghai, China), according to the manufacturer’s instructions. In preparation, a protein removal reagent was used to dissolve the cell sample. Then, the samples were subjected to two rapid freeze thaw cycles using liquid nitrogen and a 37 °C water bath. After incubation and centrifugation, the supernatant was used for further detection. To measure total glutathione, samples or standard were mixed with a working assay buffer in a 96-well plate and incubated for 5 min at 25 °C. Then 50 μL 0.5 mg/mL NADPH solution was added into each well. The final mix was measured with a microplate reader with 412 nm at 0 and 25 min. The Δ412 was calculated to measure the total glutathione concentration. For GSSG measurements, the GSH removal agent was added to the samples and standard in advance in order to remove the reduced glutathione. The processed samples and the standard were measured as described earlier. The Δ412 was calculated to measure the GSSG concentration. The GSH concentration is the concentration of total glutathione minus 2 multiplied by the GSSG concentration. The GSH/GSSG ratio was calculated afterwards. 

### 4.11. Small Interfering RNA (siRNA) Transfection

Next, 30 nM small interfering negative controls and siRNAs were added per well, based on the manufacturer’s instruction of Pepmute Transfection Reagent (Signagen, Rockville, MD, USA) after the SCC1 and SCC25 cells were seeded into 6-well plates and cultured for 24 h. RNA and protein extraction were conducted after 48 and 72 h, respectively. SiRNA sequences are listed as follows: siFTO #1: 5′-ACACUUGGCUCCCUUAUCUTT-3′; siFTO #2: 5′-GUGGCAGUGUACAGUUAUATT-3′.

### 4.12. RNA Stability Assays

To assess RNA stability in stable FTO-overexpressing cells, we treated SCC1 and SCC25 cells with actinomycin D, bringing the final concentration to 5 µg/mL. The cells were collected at 0, 6, and 12 h after incubation. The total RNA was extracted for RT-qPCR to quantify the isotopic abundance ratio of ACSL3 and GPX4 (relative to 0 h). The half-life (t1/2) reflecting the time required for mRNA expression to decrease by half was calculated.

### 4.13. Dual-Luciferase Reporter Assay

The dual-luciferase assay was performed using the Dual-Luciferase Reporter Gene Assay Kit (Yeasen, Shanghai, China), according to the manufacturer’s instructions. The m^6^A potential sites of ACSL3 and GPX4 were calculated using the SRAMP database, and only m^6^A sites with ‘very high confidence’ scores were chosen. The plasmid pmirGLO was used as the dual-luciferase vector. Each cell line was transfected with pmirGLO plasma containing wild-type or mutant ACSL3 or GPX4 using lipofectamine 3000 in a 24-well plate. After transfection for 48 h, the cells were lysed, collected, and assessed using the Dual-Luciferase Reporter Gene Assay Kit (Yeason, Shanghai, China). The lysate was centrifuged at 12,000× *g* for 1 min at 4 °C. A white opaque 96-well plate was used for the experiment, and 20 μL of the supernatant was added per well. Then, 100 μL firefly luciferase working solution was added to each well, and firefly luciferase activity was detected immediately by a microplate reader. After detecting firefly luciferase activity, a 100 μL renilla luciferase working solution was added to each well to detect the renilla luciferase activity. Renilla luciferase activity was used to normalize firefly luciferase activity to evaluate the reporter translation efficiency.

### 4.14. Methylated RNA Immunoprecipitation

A MeRIP assay was performed with an N6-Methylated RNA Immunoprecipitation (MeRIP) Kit (BersinBio, Guangzhou, China) according to the given guidelines. In general, fragmentation buffer was used to fragment total RNA into 300 nt for 2 min in a 94 °C water bath, and ethylenediamine tetra acetic acid (EDTA) was added to stop the reaction. Of the processed RNA, 1/9 was saved as input, and immunoprecipitation was applied to the rest of the RNA with a 4 μg m^6^A primary antibody at 4 °C for 4 h. The magnetic beads of protein A/G were added into the IP mix for precipitation at 4 °C for 1 h. The elution buffer and proteinase K were used to elute RNA in immunoprecipitations. The precipitated and input RNA was extracted with a phenol–chloroform–isoamylol mix. Glycogen and sodium acetate were applied to precipitate RNA at −20 °C for 3 h. The purified RNA was used for complementary DNA synthesis. The m^6^A status of ACSL3 and GPX4 was measured via RT-qPCR using the m^6^A site primers (Table 1).

### 4.15. Statistical Analysis

The mean and the standard deviation of data were analyzed using GraphPad Prism 9.0. software. The Shapiro–Wilk test was used to test normal distribution. Statistical differences between the two groups were analyzed using a two-tailed unpaired Student’s t-test, and one-way ANOVA was conducted for multiple groups. A Mann–Whitney test was performed to evaluate the FPI difference between the tumor samples and normal samples in the TCGA–OSCC cohort and GSE30784 data set. The correlation between FPI and m^6^A-related regulators was analyzed using Pearson’s correlation. A *p*-value of < 0.05 was considered statistically significant.

## Figures and Tables

**Table 1:** Primer sequences for RT-qPCR.

Gene	Primer Sequence
FTO	Forward: 5′-ACTTGGCTCCCTTATCTGACC-3′
	Reverse: 5′-TGTGCAGTGTGAGAAAGGCTT-3′
ACSL3	Forward: 5′-GCCGAGTGGATGATAGCTGC-3′
	Reverse: 5′-ATGGCTGGACCTCCTAGAGTG-3′
GPX4	Forward: 5′-GAGGCAAGACCGAAGTAAACTAC-3′
	Reverse: 5′-CCGAACTGGTTACACGGGAA-3′
PTGS2	Forward: 5′-CTGGCGCTCAGCCATACAG-3′
	Reverse: 5′-CGCACTTATACTGGTCAAATCCC-3′
β-ACTIN	Forward: 5′-CTACCTCATGAAGATCCTCACCGA-3′
	Reverse: 5′-TTCTCCTTAATGTCACGCACGATT-3′
ACSL3 m^6^A	Forward: 5′-GACCCCTGAAACTGGTCTGG-3′
	Reverse: 5′-CCTTGCAGCTTGAGACATGC-3′
GPX4 m^6^A	Forward: 5′-TGGGGCAGACCCGAAAATC-3′
	Reverse: 5′-TATTCCCACAAGGTAGCCAGG-3′

**Table 2:** Primary antibodies for Western blot.

Antibody	Dilution	Manufacturer
anti-ACSL3 primary antibody	1:2500	Abcam, UK
anti-FTO primary antibody	1:5000	Abcam, UK
anti-GPX4 primary antibody	1:3000	Abcam, UK
anti-4-HNE primary antibody	1:2500	Abcam, UK
anti-GAPDH primary antibody	1:1000	Servicebio, China

## Data Availability

The data that support the findings of this study are openly available as described in the main text.

## Supplementary Materials

The following supporting information can be downloaded at: https://www.mdpi.com/article/10.3390/ijms242216339/s1.

## Abbreviations

Oral squamous cell carcinoma	OSCC
N6-methyladenosine	m^6^A
Reactive oxygen species	ROS
Ferroptosis potential index	FPI
The Cancer Genome Atlas	TCGA
4-hydroxynonenal	4-HNE
Real-time quantity-PCR	RT-qPCR
Glutathione	GSH
Immunohistochemistry	IHC
N6-methylated RNA immunoprecipitation	MeRIP
Lung adenocarcinoma	LUAD
Polyunsaturated fatty acids	PUFAs
Monounsaturated fatty acids	MUFAs
Small interfering RNA	siRNA
