# Supplementary material for: FTO Sensitizes Oral Squamous Cell Carcinoma to Ferroptosis via Suppressing ACSL3 and GPX4
Source: Int J Mol Sci. 2023 Nov 15;24(22):16339. doi: 10.3390/ijms242216339 (PMC10671523; doi:10.3390/ijms242216339)
Supplement: Supplementary file 1 [file ijms-24-16339-s001.zip › ijms-2653198-supplementary.pdf]

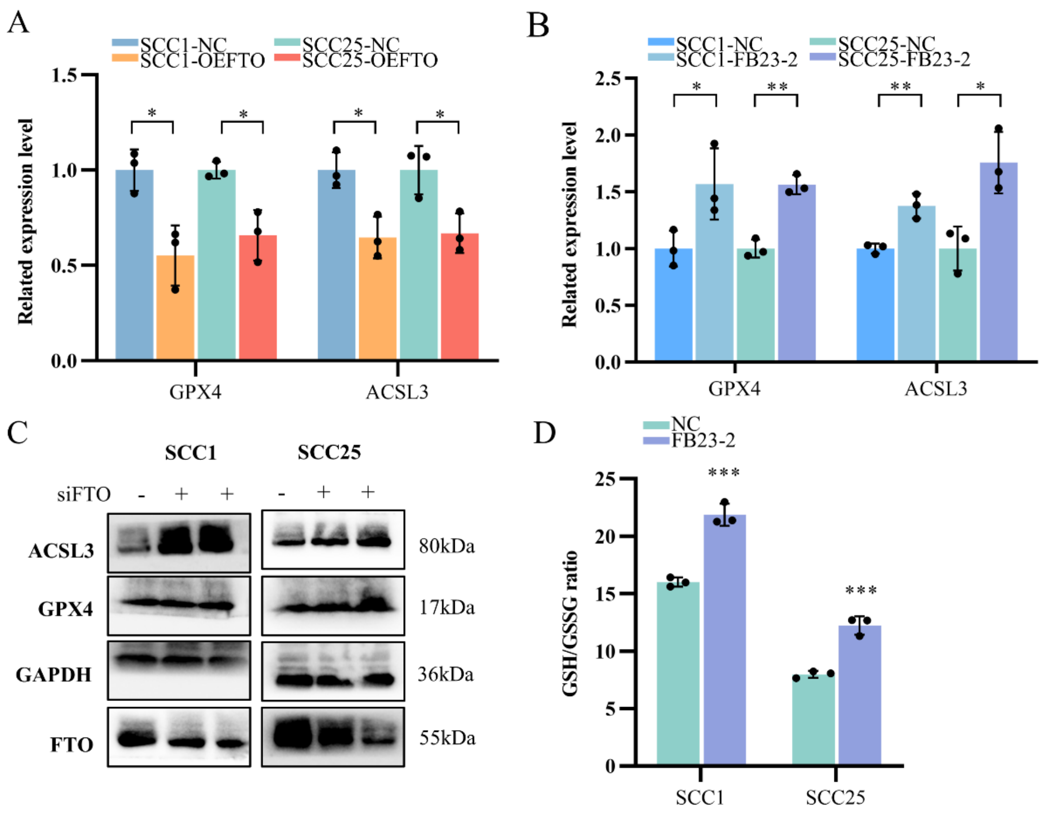

Supplementary Figure S1 FTO promotes ferroptosis in OSCC cells by downgrading the expression of ACSL3 and GPX4. (A) Statistical analysis of ACSL3 and GPX4 western blot in UM-SCC1 and SCC25 cells with or without FTO overexpression. *p < 0.05. (B) Statistical analysis of ACSL3 and GPX4 western blot in UM-SCC1 and SCC25 cells with or without FB23-2. *p < 0.05, **p < 0.01. (C) Western blot was conducted to detect the expression level of ACSL3 and GPX4 in FTO silence UM-SCC1 and SCC25 cells. (D) GSH/GSSG ratio of UM-SCC1 and SCC25 cells with or without FB23-2 treated. ***p < 0.001

A

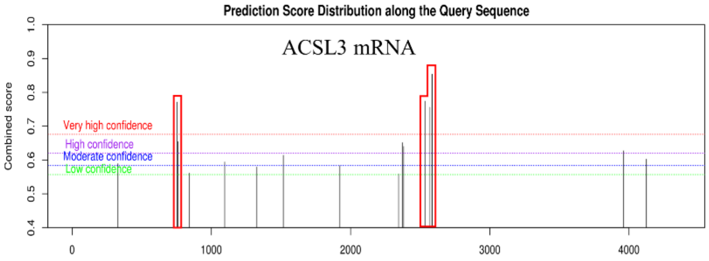

ACSL3-CDS-site1 ...AAGAACAAA...GGTGACAGA...TAAAACACA...GCGGACATT...
ACSL3-CDS-site2 ...AAGAACAAA...GGTGACAGA...TAAAACACA...GCGGACATT...
ACSL3-CDS-site3 ...AAGAACAAA...GGTGACAGA...TAAAACACA...GCGGACATT...
ACSL3-CDS-site4 ...AAGAACAAA...GGTGACAGA...TAAAACACA...GCGGACATT...

B

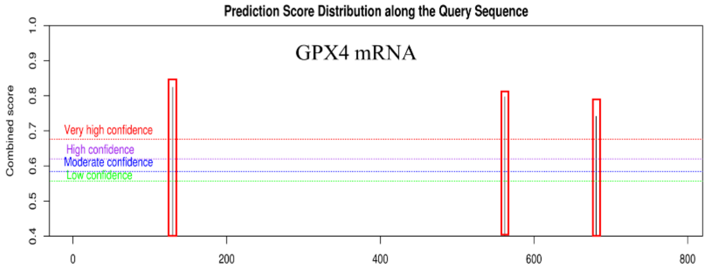

GPX4-CDS-site1 ...AAGGACATC...AAGGACCTG...GCAGACCCG...
GPX4-CDS-site2 ...AAGGACATC...AAGGACCTG...GCAGACCCG...
GPX4-3'UTR-site3 ...AAGGACATC...AAGGACCTG...GCAGACCCG...

Supplementary Figure S2 FTO demethylates the m6A modification on the mRNA of ACSL3 and GPX4. (A) The potential site and their sequence of m6A modification on ACSL3 mRNA predicted by SRAMP database. Red frame indicated the 'very high confidence' site of ACSL3 mRNA. The predicted m6A motifs were highlighted in red, respectively, and the underlined characters represented the potential m^6A modifications. (B) The potential site and their sequence of m6A modification on GPX4 mRNA predicted by SRAMP database. Red frame showed the 'very high confidence' site of GPX4 mRNA. The predicted m^6A motifs were highlighted in red, respectively, and the underlined characters represented the potential m^6A modifications.
